# Portable multi-parametric microscopy for noninvasive metabolic and vascular imaging of orthotopic tongue cancer models *in vivo*

**DOI:** 10.1117/1.JBO.30.S2.S23905

**Published:** 2025-04-23

**Authors:** Pranto Soumik Saha, Jing Yan, Caigang Zhu

**Affiliations:** University of Kentucky, Department of Biomedical Engineering, Lexington, Kentucky, United States

**Keywords:** optical metabolic imaging, tumor metabolism, hypoxia, orthotopic tongue tumors

## Abstract

**Significance:**

Precise imaging of tumor metabolism with its vascular microenvironment becomes emerging critical for cancer research because increasing evidence shows that the key attribute that allows a tumor to survive therapies is metabolic and vascular reprogramming. However, there are surprisingly few imaging techniques available to provide a systems-level view of tumor metabolism and vasculature *in vivo* on small animals for cancer discoveries.

**Aim:**

We aim to develop a new multi-parametric microscope that can faithfully recapitulate *in vivo* metabolic and vascular changes with a wide field of view and microscope-level resolution to advance cancer-related investigations. To maximize the ease and accessibility of obtaining *in vivo* tissue metabolism and vasculature measurements, we aim to develop our new metabolic imaging tool with minimal cost and size, allowing one to easily quantify tissue metabolic and vascular endpoints together *in vivo*, advancing many critical biomedical inquiries.

**Approach:**

We have combined fluorescence microscopy and dark-field microscopy in a re-emission geometry into one portable microscope to image the key metabolic and vascular endpoints on the same tissue site. The portable microscope was first characterized by tissue-mimicking phantoms. Then the multi-parametric system was demonstrated on small animals to image glucose uptake (using 2-NBDG) and mitochondrial membrane potential (using TMRE) along with vascular parameters (oxygen saturation and hemoglobin contents) of orthotopic tongue tumors *in vivo*.

**Results:**

Our phantom studies demonstrated the capability of the portable microscope for effective measurements of several key vascular and metabolic parameters with a comparable accuracy compared with our former reported benchtop spectroscopy and imaging systems. Our *in vivo* animal studies revealed increased glucose uptake and mitochondrial membrane potential along with reduced vascular oxygenation in tongue tumors compared with normal tongue tissues. The spatial analysis of metabolic and vascular images showed a more heterogeneous metabolic and oxygenation profile in tongue tumors compared with normal tongue tissues.

**Conclusions:**

Our *in vivo* animal studies demonstrated the capability of our portable multi-parametric microscope for imaging the key metabolic and vascular parameters at the same tissue site with about one hour delay using an orthotopic tongue tumor model *in vivo*. Our study showed the potential of a portable functional microscope to noninvasively evaluate tumor biology using orthotopic tongue cancer models for future head and neck cancer research.

## 1 Introduction

Metabolic reprogramming is recognized as a key hallmark of cancer,^1^^,^^2^ enabling cancer cells to survive, grow, and produce energy in hostile environments.^3^^–^^5^ In addition, variations in local vascular oxygenation levels can play a crucial role in determining the metabolic phenotype of cancer cells within the tumor.^6^^,^^7^ New evidence showed that cancer cells can evolve and adopt alternative compensatory metabolic and vascular adaptations to evade cell death, which contributes to treatment resistance and recurrence.^8^^–^^10^ Thus, targeting a single metabolic or vascular endpoint may not be sufficient to fully comprehend tumor function, nor to improve the current clinical scenario of therapy resistance and recurrence.^11^ However, there are surprisingly few techniques available that can provide a system-level view of tumor metabolism and vasculature *in vivo*, at the microscope level, in a noninvasive, and easy-to-access manner.

Positron emission tomography (PET) uses radiotracers such as fluorodeoxyglucose [F18-FDG] for measuring glucose uptake^12^ and fluoromisonidazole [F18-FMISO] for assessing tumor hypoxia,^13^ but it can quantify only one endpoint at a time and requires extensive radioactive waste disposal.^14^ Magnetic resonance imaging and magnetic resonance spectral imaging are used clinically to report on mitochondrial metabolism and glycolysis *in vivo*, but they have relatively low sensitivity.^15^^,^^16^ The Seahorse Assay reports glycolysis and oxidative phosphorylation (OXPHOS) by measuring extracellular acidification rate (ECAR) and oxygen consumption rate on *in vitro* cells but not for *in vivo* cancer screenings.^16^^,^^17^ Metabolomics can identify and quantify large numbers of metabolites in biological samples, but it does not provide information on tissue oxygenation levels.^18^^,^^19^ Immunohistochemistry can provide insights into various metabolic and vascular parameters *in vitro*, such as hypoxia (using pimonidazole)^20^ and glucose uptake (via GLUT-1 detection).^21^^,^^22^ Because of these limitations, none of these methods can provide a comprehensive picture of metabolic and vascular parameters *in vivo* with high resolution in real time. In addition, these technologies are often housed in core facilities, requiring sample or animal transport, are expensive, expertise-dependent, and involve time-consuming preparation. All these challenges limit cancer researchers’ access to high-frequency measurements. Therefore, developing new metabolic tools with portable and low-cost footprints^23^ is critical for easy-to-access, real-time measurement of key metabolic and vascular endpoints^24^^,^^25^ to advance cancer research.

Optical imaging has been widely used to measure the fluorescence ratio of endogenous co-enzymes nicotinamide adenine dinucleotide and flavin adenine dinucleotide (FAD), indirectly to report glycolysis and OXPHOS.^26^^–^^28^ This label-free, non-destructive method has been applied to cells,^29^ organoids,^30^ and *in vivo* animal models^31^ to assess metabolism. Our group has utilized the fluorescent deoxyglucose analog 2-NBDG to study glucose uptake in both cell and animal models, validating it as a reliable glucose uptake marker.^32^^–^^34^ Though 2-NBDG is a fluorescent derivative of fluorodeoxyglucose (FDG) used in PET imaging, it offers the advantage of measuring glucose uptake kinetics at multiple time points. By contrast, FDG provides information uptake at only a single time point.^35^ We have also used cationic dye tetramethylrhodamine, ethyl ester (TMRE) to report on mitochondrial membrane potential (MMP), which has also been validated with numerous studies.^32^^,^^36^^–^^38^ We have also developed a novel staggered delivery strategy that enables near-simultaneous imaging of 2-NBDG and TMRE fluorescence probes without any chemical or optical crosstalk.^39^^,^^40^ Optical imaging has also proven to be a valuable tool for investigating tumor hemodynamics, particularly in understanding the effects of tumor hypoxia.^41^ Unlike polarographic microelectrodes, which are a well-established but invasive method for measuring tissue ${\mathrm{pO}}_{2}$ at the microvascular level, optical imaging offers a noninvasive and label-free approach.^42^ Optical imaging allows for the spatial mapping of tumor hypoxia without causing any tissue damage.

In this study, we reported a portable multi-parametric intravital microscope for noninvasive metabolic and vascular imaging of biological tissues. The system integrates the functions of a fluorescent microscope with metabolic probes (2-NBDG and TMRE) and dark-field-based diffuse reflectance imaging to concurrently monitor glucose uptake, MMP, and vascular parameters [oxygenation (${\mathrm{StO}}_{2}$) and hemoglobin contents]. To characterize the system and image processing algorithms for vascular and metabolic parameter quantification, we have conducted several tissue-mimicking phantom studies for both fluorescence imaging and dark-field-based diffuse reflectance imaging. Our fluorescence phantom studies showed that the new portable and cost-effective system had a comparable sensitivity compared with our former reported expensive benchtop microscope^39^ for the measurement of the two metabolic probes. Our hemoglobin phantom studies showed that our portable microscope had a comparable accuracy compared with our well-established spectroscopy system^43^ for the estimations of ${\mathrm{StO}}_{2}$ and total hemoglobin contents. To demonstrate the capabilities of our microscope for cancer imaging, we performed *in vivo* imaging of orthotopic tongue tumors to study metabolism and vasculature. The use of the orthotopic tongue tumor model provides a more accurate representation of the HNC tumor microenvironment.^44^ To our knowledge, no other studies have conducted *in vivo* metabolic and vascular imaging on this model before. From our *in vivo* animal studies, we observed lower ${\mathrm{StO}}_{2}$ in tongue tumors compared with normal tissue, alongside higher MMP and increased glucose uptake in the tongue tumors. Spatial analysis of the metabolic and vascular images further revealed a more diverse metabolic profile in tongue tumors compared with normal tongue tissues. All of our findings demonstrated the ability of our portable multi-parametric microscope to capture the key metabolic and vascular parameters on the same tissue site with about one hour delay, offering a new way for cancer studies using small tumor models. By obtaining such multi-dimensional insights into the metabolic and vascular landscape of tumors, our platform may contribute significantly to cancer research that will further improve patient outcomes in cancer care.

## 2 Materials and Methods

### 2.1 Portable Multi-parametric Functional Microscope

**Fig. 1:**
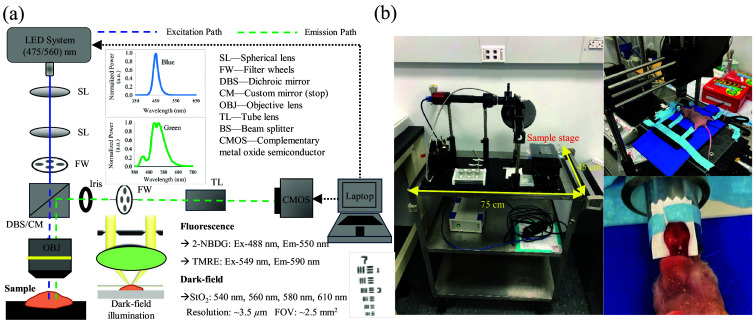
(a) Schematic diagram of the portable multi-parametric microscopy system, (b) photo of the actual system in a stand-alone portable cart, and its potential use for *in vivo* mice tongue imaging. The total cost for the critical optical components is less than $10k.

To minimize the system size, a dark-field microscope^45^ and a fluorescence microscope were integrated into one single portable platform using the layout in Fig. 1(A) to perform fluorescence imaging and dark-field-based diffuse reflectance imaging in re-emission mode. To minimize the cost, a high-power dual-color LED (450SR-545SR, Prizmatix) was utilized instead of expensive lasers for both fluorescence imaging and dark-field-based diffuse reflectance imaging. Light from the LED was coupled into an optical fiber (NA = 0.39, SM93L01, Thorlabs, Newton, United States) using a spherical lens (LA1805, Thorlabs) and then collimated with a second spherical lens (LA1951, Thorlabs). The collimated light was further directed to the objective lens (4X, MRP70040, Nikon, Tokyo, Japan) via a dichroic mirror (for fluorescence imaging) or custom-cut reflective mirror (for dark-field-based diffuse reflectance imaging). The objective lens will deliver the illumination light onto the sample and collect the reflected light into the collection channel. The custom-cut reflective mirror has an ellipse-shape hole in the center so it will shape the collimated light beam to a ring illumination beam onto the objective lens when the mirror was implemented in the wheel with a 45-deg angle relative to the incident beam. The ring beam formed by the custom-cut reflective mirror on the objective lens surface had a beam diameter of 6 mm and an inner diameter of 4 mm. In the collection end, an iris (SM1D12C, Thorlabs) of 4-mm diameter was used along with this custom-cut reflective mirror to remove the specular reflectance thereby providing dark-field-based diffuse reflectance imaging. As illustrated in Fig. 1(A), a 488-nm bandpass filter (10-nm bandpass, 65147, Edmund Optics, Barrington, New Jersey, United States) was used to generate light for 2-NBDG (glucose probe) excitation, a 549-nm bandpass filter (15-nm bandwidth, 87751, Edmund Optics) was used to generate light for TMRE (MMP probe) excitation to minimize the autofluorescence and crosstalk. A 506-nm dichroic mirror (FF506-Di03-25x36, Semrock, Rochester, New York) and a 573-nm dichroic mirror (FF573-Di01-25×36, Semrock) were placed in the beam splitter wheel for 2-NBDG and TMRE imaging, respectively. The total illumination power was measured to be 8.9 mW for 2-NBDG excitation and 8.1 mW for TMRE excitation with an illumination area of ∼3.5  mm in diameter. In the collection end, an infinity-corrected tube lens (TTL200, Thorlabs) and a compact scientific-grade CMOS monochrome camera (CS505MU, Thorlabs) were used for photon detection. For fluorescence imaging, bandpass filters at 550 nm (40 nm bandpass, FBH550-40, Thorlabs) and 600 nm (40 nm bandpass FBH600-40, Thorlabs) were used to selectively capture the emitted fluorescence signals from 2-NBDG and TMRE, respectively. Spectral tuning for dark-field-based diffuse reflectance imaging was achieved using multiple bandpass filters centered at 540 nm (65157, Edmund Optics), 560 nm (88011, Edmund Optics), 580 nm (65161, Edmund Optics), and 610 nm (65164, Edmund Optics), each with a 10 nm bandwidth. These four bandpass filters were used to enable quantitative analysis of overlapping spectra and to gather dark-field-based diffuse reflectance data for ${\mathrm{StO}}_{2}$ quantification. A laboratory jack (L490, Thorlabs) combined with an X-Y translation stage (XYT1, Thorlabs) was used as one X-Y-Z sample stage, enabling precise adjustment of the sample’s position in all three dimensions. The distance between the sample (tongue tissue) and the objective lens was determined by the objective lens focal plane (30 mm). The sample-to-objective lens distance was manually adjusted to 30 mm to capture the sharpest image possible. Once the system was established, it was packaged into a stand-alone cart (Fig. 1(B)) for point-of-care imaging. The portable microscope has a field of view of approximately $2.5\;{\mathrm{mm}}^{2}$ and a resolution of about 3.5  μm measured using a USAF 1951 resolution target.

### 2.2 Tissue-Mimicking Phantom Studies

To characterize the microscope system and image processing algorithms for vascular and metabolic parameters quantification, we have conducted various tissue-mimicking phantom studies for both fluorescence imaging and dark-field-based diffuse reflectance imaging. Specifically, fluorescence phantoms including PBS-based scattering solutions (reduced scattering levels were $5\;{\mathrm{cm}}^{-1}$, $10\;{\mathrm{cm}}^{-1}$, and $15\;{\mathrm{cm}}^{-1}$ on average between 400 and 600 nm) with 2-NBDG (0 to 8  μM) and TMRE probes (0 to 70 nM) were created to characterize the system’s sensitivity and dynamic range for quantification of the two metabolic probes at biologically relevant concentrations. On the other hand, turbid hemoglobin phantoms (a mixture of DI water, intralipid, and human hemoglobin) with various optical properties were used to characterize the microscope system for rapid estimations of ${\mathrm{StO}}_{2}$ and total hemoglobin contents. Three groups of tissue-mimicking turbid phantoms with different initial reduced scattering levels ($5\;{\mathrm{cm}}^{-1}$, $10\;{\mathrm{cm}}^{-1}$, and $15\;{\mathrm{cm}}^{-1}$ on average between 400 and 600 nm) were prepared. Within each group of phantoms, five increasing concentrations of hemoglobin were added to generate average absorption coefficients of $1.0-5.5\;{\mathrm{cm}}^{-1}$ (on average between 400 and 600 nm). Hemoglobin concentration was increased by adding aliquots of the stock hemoglobin solution with a known absorption coefficient spectrum that was determined by a spectrophotometer. After each addition of hemoglobin stock solution, both dark-field-based diffuse reflectance images at four wavelengths (using the reported microscope) and diffuse reflectance spectra (using our former reported spectroscopy^43^) were measured simultaneously from the phantoms. Due to the lack of resources, we were not able to validate the ${\mathrm{StO}}_{2}$ estimation algorithm against ${\mathrm{pO}}_{2}$ directly in our lab, whereas our well-established spectroscopy system^43^ may still serve as a proper reference for this validation study.

### 2.3 Animal Experiments

The portable multi-parametric microscope was tested to capture several key metabolic and vascular endpoints in an orthotopic tongue model, following a protocol approved by the Institutional Animal Care and Use Committee at the University of Kentucky. Ten female athymic nude mice (nu/nu, Jackson Laboratory) with an age of 8 to 10 weeks were used for the study. Animals were assigned to (1) normal group (n=5) and (2) tumor group (SCC-61; n=5). The tumor group received injections of SCC-61 cells^43^ (0.05 mL of cell solution in Matrigel with a concentration of $1.5-2\times 10^{6}\;\text{cells}/\text{animal}$) into the ventral side of the tongue. The mice were then monitored routinely for 2 weeks following tumor cell injection. The imaging study was conducted on the 10^th^ day after tumor cell injection under anesthesia. The mice breathed 1.5% (v/v) isoflurane mixed with room air during anesthesia and were placed over a heating pad to maintain body temperature. Mice’s tongue was pulled out gently using rubber-tip forceps and surgical tapes were used to fix it on a flat platform [as shown in Fig. 1(B)]. To minimize variations in metabolic demand, the mice were fasted for 6 h before conducting the optical imaging experiments. All animal imaging studies were conducted using the microscopy system described in Fig. 1. The imaging process began with the acquisition of dark-field-based diffuse reflectance images, which were captured at spectral wavelengths of 540 nm, 560 nm, 580 nm, and 610 nm. The exposure time for the diffuse reflectance imaging was set to 300 ms, with a gain setting of 10. Subsequently, for fluorescence imaging, all mice received a tail-vein injection of TMRE (100  μL of 100  μM) followed by a tail-vein injection of 2-NBDG (100  μL of 6 mM) after a delay of 20 min.^38^^,^^39^ The exposure time for fluorescence imaging was set to 1000 ms, with a gain setting of 15 for 2-NBDG and a gain setting of 25 for TMRE. Baseline fluorescence images were captured from the tissue before any injections. Fluorescence signals of TMRE and 2-NBDG were monitored for 80 min and 60 min, respectively, in a dark room to minimize interference from ambient light.

### 2.4 Data Processing to Extract Vascular and Metabolic Parameters

#### 2.4.1 Vascular parameters extraction from dark-field-based diffuse reflectance images

**Fig. 2:**
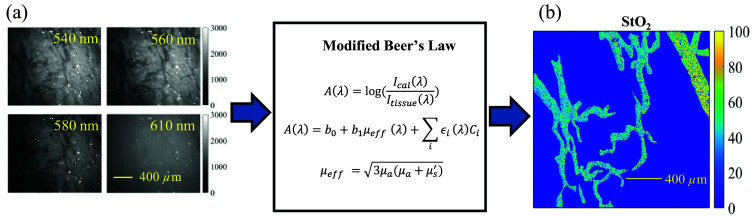
Typical dark-field-based diffuse reflectance images of mice tongue (a) captured at 540 nm, 560 nm, 580 nm, and 610 nm. A typical hemoglobin oxygen saturation (${\mathrm{StO}}_{2}$) map (b) estimated using the modified Beer’s law. The scale bar represents 400  μm.

Dark field microscopy combined with a Gabor-filter-based image processing method has been explored for automatic vasculature structure quantification to study tumor angiogenesis,^45^ whereas our study primarily focused on tumor metabolism and hypoxia. Therefore, we did not employ the published automatic vessel segmentation method for vascular features analysis. Rather, we relied on manual segmentation of the vessels to ensure the best accuracy for functional vascular parameters estimation. Specifically, hand-traced masks of the vessels were created from the dark-field-based diffuse reflectance images using the “Image Segmenter” toolbox in MATLAB software to ensure precise vessel identification for ${\mathrm{StO}}_{2}$ and hemoglobin concentration estimation. Dark-field-based diffuse reflectance images were calibrated using a 20% reflectance standard (Spectralon, Labsphere, North Sutton, New Hampshire). The vascular parameters including ${\mathrm{StO}}_{2}$ and total hemoglobin indicator (THb) were extracted from the dark-field-based diffuse reflectance images using the method described in Fig. 2. Specifically, the dark-field-based diffuse reflectance images were processed using a modified version of Beer’s Law to quantify ${\mathrm{StO}}_{2}$ as reported previously.^46^^,^^47^ This method models tissue absorption as a linear combination of absorption and scattering components. Tissue absorption A(λ) for a particular wavelength λ can be written as

$$A(\lambda )=\log(\frac{I_{\mathrm{cal}}(\lambda )}{I_{\text{tissue}}(\lambda )})=b_{0}+b_{1}\mu_{\mathrm{eff}}(\lambda )+\underset{i}{\sum }\varepsilon_{i}(\lambda )C_{i},$$ (1)

where $I_{\mathrm{cal}}$ represents the calibrated reflective standard intensity and $I_{\text{tissue}}$ is diffuse reflectance measured on tissues. The constant term $b_{0}$ accounts for the variability of the light source intensity. $\mu_{\mathrm{eff}}$ (λ) is the effective attenuation coefficient, calculated based on the optical properties of mice tongue tissue,^43^ and $b_{1}$ serves as an adjustment factor for the effective attenuation coefficient. $\varepsilon_{i}(\lambda )$ is the molar extinction coefficient of the dominant absorbers, oxyhemoglobin ($\varepsilon_{\mathrm{HbO}2}$), and deoxyhemoglobin ($\varepsilon_{\mathrm{Hb}}$). $C_{i}(\lambda )$ represents the product of the concentration of these absorbers and the effective path length through the tissue. Dark-field-based images of mice tongues were captured at four wavelengths [Fig. 2(A)], corresponding to key absorption peaks in oxyhemoglobin (${\mathrm{HbO}}_{2}$) and deoxyhemoglobin (Hb) spectra. A non-negative least squares fit was applied to determine the values of $b_{0}$, $b_{1}$, $C_{\mathrm{Hb}}$, and $C_{\mathrm{HbO}2}$ from the diffuse reflectance spectral images. The solution was accepted or rejected based on an $R^{2}$ value threshold of 0.9. The total hemoglobin indicator (THb) was determined as the sum of $C_{\mathrm{HbO}2}$ and $C_{\mathrm{Hb}}$, whereas the ${\mathrm{StO}}_{2}$ was calculated as the ratio of $C_{\mathrm{HbO}2}$ and $C_{\text{THb}}$. Using these extracted parameters, a ${\mathrm{StO}}_{2}$ or THb map of the mice tongue image was generated (Fig. 2(B)). To analyze the distribution of ${\mathrm{StO}}_{2}$ or THb, a kernel smoothing function (ksdensity) was applied to generate pixel-level probability distributions for both ${\mathrm{StO}}_{2}$ or THb maps. It should be noted that this method implemented in a microscope system for ${\mathrm{StO}}_{2}$ estimation has been well validated against ${\mathrm{pO}}_{2}$ using blood smears by others before.^46^^,^^47^ To further validate this method using our own microscope, we have conducted tissue-mimicking phantom studies to validate the algorithm using our well-established spectroscopy system^43^ for accurate estimations of ${\mathrm{StO}}_{2}$ and total hemoglobin contents.

#### 2.4.2 Metabolic Parameters Extraction

Metabolic parameters were extracted from the fluorescence images of 2-NBDG and TMRE uptake. For fluorescence image analysis, all images were background subtracted to ensure an accurate assessment of the 2-NBDG and TMRE signals. The tissue absorption primarily from the vessel region can alter the fluorescence signal, leading to variations in the measured intensities. Therefore, we have excluded these blood vessel regions during the quantitative analysis in our study to reflect only the 2-NBDG and TMRE uptake in the tissue space. To exclude the blood vessels from the quantitative analysis, a manually traced blood vessel mask was applied to each set of fluorescence images. The blood vessel mask was generated from both dark-field-based diffuse reflectance images (for major blood vessels) and fluorescence images (for small vessels). Only the tissue regions without blood vessels were considered for fluorescence intensity calculation of either TMRE or 2-NBDG. The average intensity values of the non-blood vessel tissue regions at every time point were calculated to generate a time course kinetic curve. The final 2-NBDG and TMRE uptake were calculated using the same time points: 2-NBDG uptake 60 min after 2-NBDG injection ($2\text{-}{\mathrm{NBDG}}_{60}$) and TMRE uptake 80 min after TMRE injection (${\mathrm{TMRE}}_{80}$). There was a 20-min delay between TMRE injection and 2-NBDG injection; thus, the $2\text{-}{\mathrm{NBDG}}_{60}$ and ${\mathrm{TMRE}}_{80}$ uptake were measured at the same time point. To analyze the distribution of metabolic activity, a kernel smoothing function (ksdensity) was applied to generate pixel-level probability distributions for both 2-NBDG and TMRE images.

#### 2.4.3 Spatial heterogeneity and correlation analysis

To assess the spatial heterogeneity of metabolism in the extracted fluorescence images, two approaches were utilized: local range variation analysis and intensity-based clustering. The local range variation analysis measures the localized variability in metabolic activities by calculating the local range intensity variation for each pixel in an image as a new way to quantify the metabolism heterogeneity as introduced previously.^48^ Specifically, a “sliding window” of a 3×3 neighborhood (9 pixels, ∼13  μm*13  μm area) was used in fluorescence image processing to compute the intensity difference between the central analyzed pixel and its neighborhood maximum. The 3×3 is the smallest window size for this image processing, whereas a larger window size can also be used for the same purpose but with decreased sensitivity for local variation detection. A larger local variation value indicates a higher heterogeneity for the analyzed pixel.^48^ To illustrate the cell metabolic activities and metabolism heterogeneity with more details, a clustering approach based on pixel intensity levels^48^ was also used for metabolic image processing. Specifically, each pixel of the $2\text{-}{\mathrm{NBDG}}_{60}$ or ${\mathrm{TMRE}}_{80}$ images was clustered into a high or low pixel ($2\text{-}{\mathrm{NBDG}}_{60}$ or ${\mathrm{TMRE}}_{80}$) by comparing the pixel’s intensity with its corresponding global average intensity of $2\text{-}{\mathrm{NBDG}}_{60}$ or ${\mathrm{TMRE}}_{80}$ for this animal. Then, the paired $2\text{-}{\mathrm{NBDG}}_{60}$ and ${\mathrm{TMRE}}_{80}$ images were merged and further clustered into four groups: low 2-NBDG and low TMRE group, low 2-NBDG and high TMRE group, high 2-NBDG and low TMRE group, and high 2-NBDG and high TMRE group, which were coded as 1, 2, 3, and 4, respectively.

The measurement of key vascular and metabolic endpoints on the same tissue site enables us to investigate the potential correlations between tumor metabolism and the associated vasculature. The direct evaluation of the correlations between two images should be done in a pixel-by-pixel way, whereas it is difficult to use this pixel-by-pixel approach for the parameter pairs that are not pixel-by-pixel matched, such as the vascular parameters (${\mathrm{StO}}_{2}$ and THb) from vessel regions versus metabolic parameters ($2\text{-}{\mathrm{NBDG}}_{60}$ and ${\mathrm{TMRE}}_{80}$) from non-vessel regions. Therefore, we have decided to use a regional average approach, i.e., dividing each image into several equal regions as a practical way to explore the correlations among different parameter pairs. The number of equal regions one image is divided into should be balanced to ensure (1) each subregion can represent sufficient heterogeneity (or variability) and (2) each subregion should have both sufficient vascular and metabolic information for the correlation analysis. Taken together, we divided each pair of vascular and metabolic images into nine (3×3) equal nonants, then we explored the correlations between the parameter pairs based on the nine nonant-matched mean values per animal for all animals. In this way, we can take advantage of imaging capability to explore the regional correlations between vascular parameters and metabolic parameters but with an equal presentation. A Student’s t-test was used for all statistical analyses, with a p-value of less than 0.05 considered statistically significant. Data processing and statistical analysis were conducted using MATLAB 2023a (MathWorks, United States).

## 3 Results

### 3.1 Phantom Study Results

**Fig. 3:**
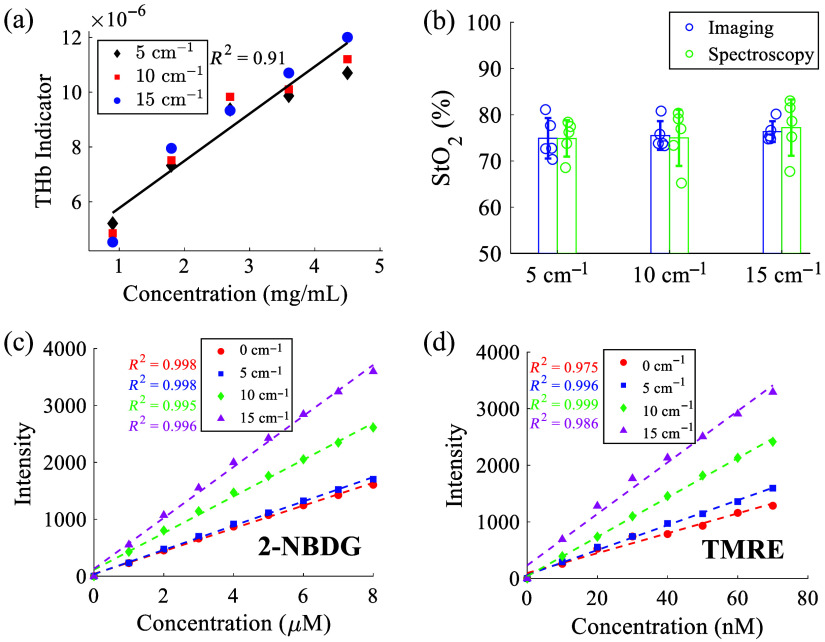
(a) Comparison of the microscope-extracted THb indicator with expected THb concentrations. (b) The ${\mathrm{StO}}_{2}$ values estimated by the microscope and our well-established spectroscopy for tissue-mimicking phantoms with various reduced scattering (averaged reduced scattering were $5\;{\mathrm{cm}}^{-1}$, $10\;{\mathrm{cm}}^{-1}$, and $15\;{\mathrm{cm}}^{-1}$) and absorption levels (1.0 to $5.5\;{\mathrm{cm}}^{-1}$). Fluorescence microscope for sensitive imaging of 2-NBDG (c) and TMRE (d) solutions in PBS-based scattering media (averaged reduced scattering were $5\;{\mathrm{cm}}^{-1}$, $10\;{\mathrm{cm}}^{-1}$, and $15\;{\mathrm{cm}}^{-1}$) at biologically relevant concentrations. The integration time for fluorescence phantoms was set to be 250 ms for both 2-NBDG and TMRE, whereas the gain values for 2-NBDG and TMRE imaging were set to be the same as those used in animal imaging.

Figure 3 shows the performance of our new portable optical microscope for fluorescence probe measurement and vascular parameter estimation characterized via tissue-mimicking phantom studies. Figure 3(A) shows the comparison between the microscope-measured THb indicator and the corresponding expected THb concentrations. As illustrated, the microscope along with the simple image processing algorithm can effectively indicate the THb with high performance ($R^{2}$ is above 0.9) from the tissue-mimicking phantoms. Figure 3(B) shows ${\mathrm{StO}}_{2}$ values estimated using the microscope system and our formerly well-established spectroscopy system^43^ for all tissue-mimicking phantoms with various optical properties. The ${\mathrm{StO}}_{2}$ values estimated by the microscope are comparable to the ${\mathrm{StO}}_{2}$ values estimated by the spectroscopy platform for phantoms with various scattering levels. Figures 3(C) and 3(D) show the performance of the microscope for fluorescence measurement of the two metabolic probes at biologically relevant concentrations. A linear correlation between fluorescence intensity and fluorophore concentration was observed for each fluorophore as expected ($R^{2}$ values are above 0.975 for both 2-NBDG and TMRE). The fluorescence phantom studies showed that our new portable microscope had a comparable sensitivity compared with our former published expensive benchtop microscope^39^ for the measurement of the two metabolic probes.

### 3.2 Vascular Parameters of Normal Tongues and Tumors

**Fig. 4:**
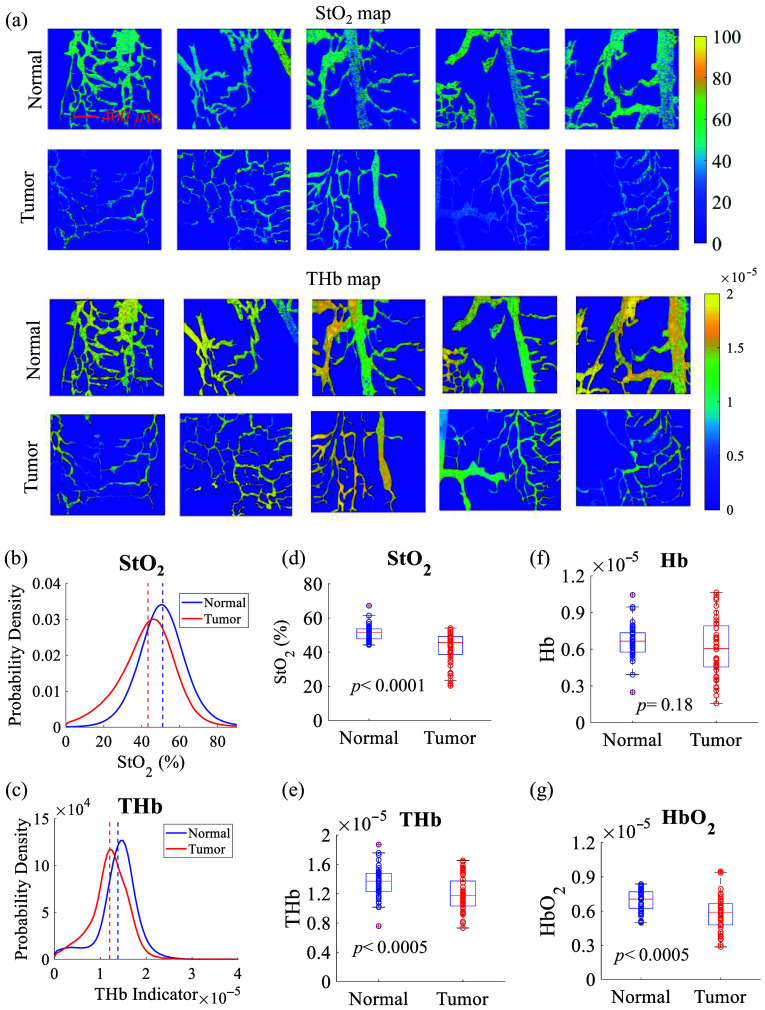
Vessel map images of vascular ${\mathrm{StO}}_{2}$ and THb indicator of all five normal tongue tissues and five tongue tumor tissues. Kernel distribution of pixel intensities for (b) ${\mathrm{StO}}_{2}$ and (c) THb indicator for normal tongue tissues and tongue tumors. Dashed lines indicate the mean locations. Regionally averaged vascular parameters obtained from vascular images for all animals including (d) ${\mathrm{StO}}_{2}$, (e) THb indicator, (f) Hb indicator, and (g) ${\mathrm{HbO}}_{2}$ indicator. The regional average approach introduced in the method section was used to represent each set of animal data with nine mean values for the boxplots. A Student’s t-test was used for all statistical analyses. The scale bar represents 400  μm.

The vascular parameters obtained from dark-field-based diffuse reflectance images of normal and tumor tongues are shown in Fig. 4. Vessel maps of calculated vascular ${\mathrm{StO}}_{2}$ and total hemoglobin (THb) indicator for all five normal tongue tissues and five tongue tumors are shown in Fig. 4(A). Figures 4(B) and 4(C) show pixel-level probability distributions for both ${\mathrm{StO}}_{2}$ or THb generated from all five normal tongue tissues and five tumor tissues. The pixel-level probability distributions show that tongue tumor tissues have lower ${\mathrm{StO}}_{2}$ and THb compared with normal tongue tissues, either by comparing the histogram peak locations or the mean locations (highlighted by dashed lines) between the two tissue types. To statistically compare the vascular endpoints between normal and tumor groups, the regional average approach introduced in the method section was used to represent each set of animal data with nine mean values. Figure 4(D) shows that the tumor tongue tissues have significantly lower ${\mathrm{StO}}_{2}$ compared with the normal tongue (p<0.0001), suggesting obvious hypoxia in the tumor tongue regions. Figure 4(E) shows that normal tongue tissues had a statistically higher THb indicator compared with the tongue tumors significantly (p<0.0005). Figure 4(F) shows that both normal and tumor tongue have comparable levels of Hb indicator (p=0.18). Figure 4(G) shows that tongue tumors significantly lower the ${\mathrm{HbO}}_{2}$ indicator compared with normal tongue tissue (p<0.0005). It should be noted that the THb indicator, Hb indicator, and ${\mathrm{HbO}}_{2}$ indicator do not provide precise measurements of these vascular parameters but rather an estimation of these quantities.

### 3.3 Metabolic Parameters of Normal Tongue and Tongue Tumors

**Fig. 5:**
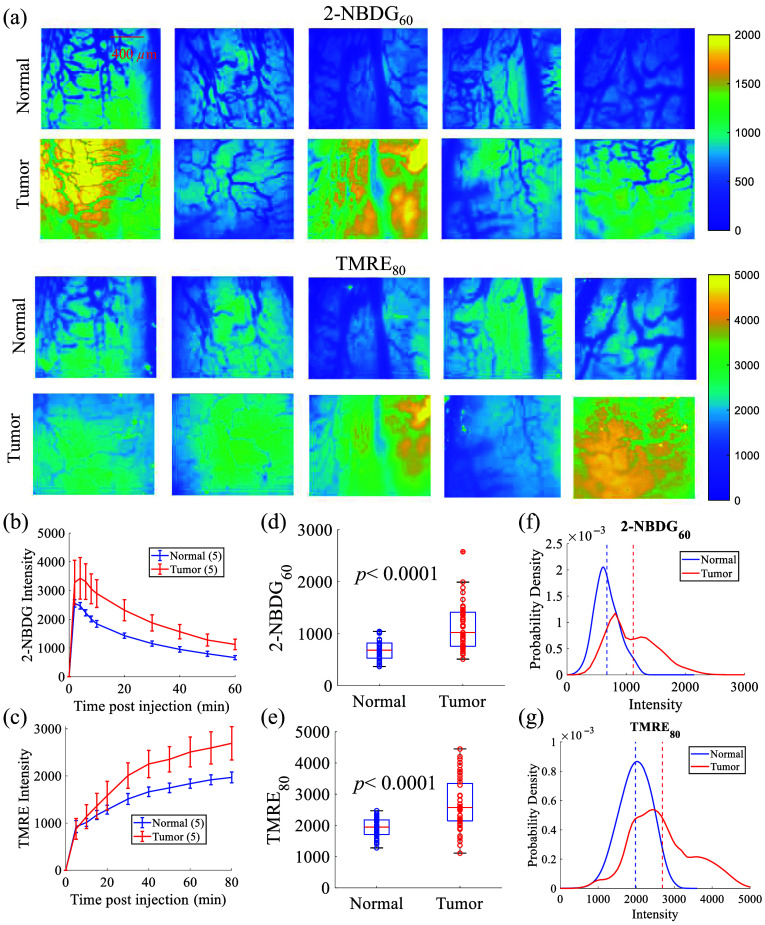
(a) Fluorescence images of 2-NBDG uptake at 60 min ($2\text{-}{\mathrm{NBDG}}_{60}$) and TMRE uptake at 80 min (${\mathrm{TMRE}}_{80}$) for all five normal tongue tissues and five tongue tumor tissues. There was a 20-min delay between TMRE injection and 2-NBDG injection; thus, the $2\text{-}{\mathrm{NBDG}}_{60}$ and ${\mathrm{TMRE}}_{80}$ uptake were measured at the same time point. (b) 2-NBDG uptake kinetics. (c) TMRE uptake kinetics. Boxplots of (d) $2\text{-}{\mathrm{NBDG}}_{60}$ and (e) ${\mathrm{TMRE}}_{80}$ for both normal and tumor groups. The regional average approach introduced in the method section was used to represent each set of animal data with nine mean values for the boxplots. Kernel distribution of pixel intensities for (f) $2\text{-}{\mathrm{NBDG}}_{60}$ and (g) ${\mathrm{TMRE}}_{80}$ for both normal tongue tissues and tongue tumor tissues. Dashed lines indicate the mean locations. The data in panels (f) and (g) were extracted from all 10 animals. All fluorescence images in panel (a) were background subtracted. A Student’s t-test was used for all statistical analyses. The scale bar represents 400  μm.

The fluorescence images of $2\text{-}{\mathrm{NBDG}}_{60}$ and ${\mathrm{TMRE}}_{80}$ of all five normal tongue tissues and five tongue tumors are shown in Fig. 5(A). The fluorescence uptake kinetics of 2-NBDG over 60 min is shown in Fig. 5(B). The 2-NBDG fluorescence reached peak intensity within 2–6 min post-injection and then gradually decreased. The tumor group is seen to exhibit higher 2-NBDG uptake compared with the normal group [Fig. 5(B)]. Similarly, Fig. 5(C) shows the TMRE fluorescence uptake kinetics over 80 min. The intensity of TMRE fluorescence gradually increased in both groups, with a steeper rise observed in the tumor group. The signal stabilized at ∼60  min post-injection for both groups, with the tumor group exhibiting a higher TMRE uptake compared with the normal tongue group. To statistically compare the metabolic endpoints between normal and tumor groups, the regional average approach introduced in the method section was used to represent each set of animal data with nine mean values. The boxplot in Fig. 5(D) shows that the $2\text{-}{\mathrm{NBDG}}_{60}$ is significantly higher in tongue tumors compared with normal tongue tissues (p<0.0001). Similarly, Fig. 5(E) shows that the ${\mathrm{TMRE}}_{80}$ is also significantly higher in tongue tumors compared with normal tongue tissues (p<0.0001). The kernel distribution of pixel intensities for $2\text{-}{\mathrm{NBDG}}_{60}$ and ${\mathrm{TMRE}}_{80}$ (based on all animal images) are shown in Figs. 5(F) and 5(G), respectively, with the dashed lines indicating the mean intensities for each group. The tongue tumors have broader intensity distributions compared with the normal group. The pixel-level probability distributions further show that tongue tumor tissues have higher $2\text{-}{\mathrm{NBDG}}_{60}$ and ${\mathrm{TMRE}}_{80}$ compared with normal tongue tissues, either by comparing the histogram peak locations or the mean locations between the two tissue types.

### 3.4 Spatial Heterogeneity of the Captured *In vivo* Metabolic Parameters

**Fig. 6:**
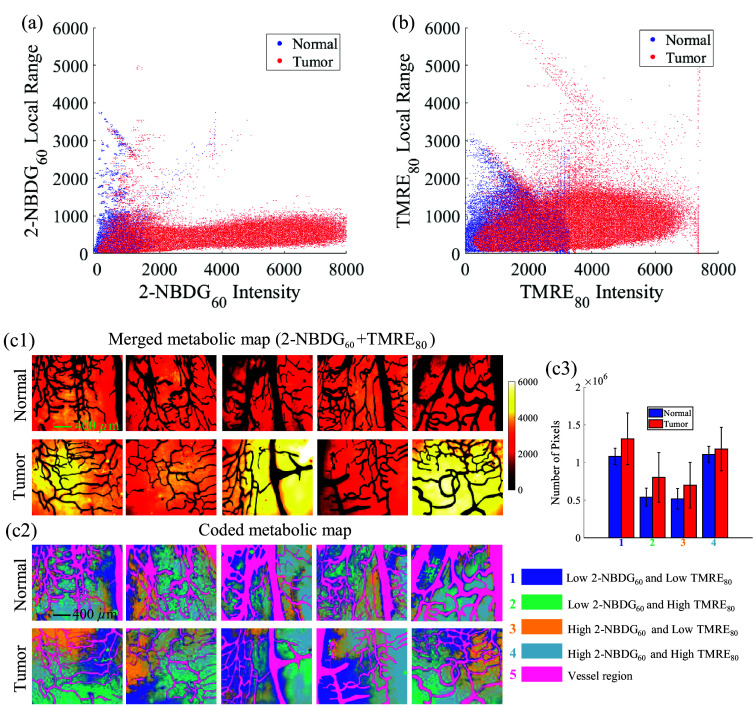
(a) Pixel-based $2\text{-}{\mathrm{NBDG}}_{60}$ fluorescence intensity distribution versus their corresponding $2\text{-}{\mathrm{NBDG}}_{60}$ local range variations. (b) Pixel-based ${\mathrm{TMRE}}_{80}$ fluorescence intensity distribution versus their corresponding ${\mathrm{TMRE}}_{80}$ local range variations. A higher local range variation in the y-axis indicates a higher heterogeneity. The data in panels (a) and (b) were extracted from all 10 animals. (c1) Merged overall metabolic heat map for all five normal tongue tissues and five tumor tissues. (c2) Clustered overall metabolic heat map for all five normal tongue tissues and five tumor tissues. (c3) Bar plot showing the number of pixels in the following groups: (1) low $2\text{-}{\mathrm{NBDG}}_{60}$ and low ${\mathrm{TMRE}}_{80}$, (2) low $2\text{-}{\mathrm{NBDG}}_{60}$ and high ${\mathrm{TMRE}}_{80}$, (3) high $2\text{-}{\mathrm{NBDG}}_{60}$ and low ${\mathrm{TMRE}}_{80}$, and (4) high $2\text{-}{\mathrm{NBDG}}_{60}$ and high ${\mathrm{TMRE}}_{80}$. The scale bar represents 400  μm.

The metabolic heterogeneity of $2\text{-}{\mathrm{NBDG}}_{60}$ and ${\mathrm{TMRE}}_{80}$ uptake was analyzed using local range variation analysis as shown in Figs. 6(A) and 6(B). In Fig. 6(A), the local range analysis of $2\text{-}{\mathrm{NBDG}}_{60}$ shows that local range variation values are more pronounced in the low-intensity pixels for both normal tissues and tongue tumors. However, in the high-intensity pixel regions, the local range values are not elevated for tongue tumors. Local range analysis of ${\mathrm{TMRE}}_{80}$ [Fig. 6(B)] shows that tongue tumors have higher local range values compared with normal tongue tissues. The local range variation analysis data shown in Figs. 6(A) and 6(B) suggested that tongue tumor tissues have a higher metabolic heterogeneity compared with normal tongue tissues.

Figure 6(C) illustrates the overall metabolic activity analysis by combining the two parameters ($2\text{-}{\mathrm{NBDG}}_{60}+{\mathrm{TMRE}}_{80}$) into one image using the previously reported clustering approach based on pixel intensity levels.^48^ Figure 6(C1) shows the merged metabolic heat map from which one can observe the metabolic activity from region to region for each animal for all animals. Figure 6(C2) shows the coded metabolic heat map using the cluster approach,^48^ from which one can capture the detailed metabolic activity contributions from 2-NBDG or TMRE from region to region for each animal for all animals. The bar plot in Fig. 6(C2) summarizes the number of pixels associated with each spatial clustering group shown in Fig. 6(C1). It shows that tongue tumors exhibit a significantly higher number of pixels for group 2 (low $2\text{-}{\mathrm{NBDG}}_{60}$ and high ${\mathrm{TMRE}}_{80}$) and group 3 (high $2\text{-}{\mathrm{NBDG}}_{60}$ and low ${\mathrm{TMRE}}_{80}$) compared with the normal tongues, indicating a more diverse metabolic profile for the tongue tumors compared with the normal tongue tissues. We also observed larger variations in the bar plots for tumor tissues compared with normal tongue tissues, which suggested a higher metabolic heterogeneity across the different tumors compared with that in normal tongue tissues.

### 3.5 Correlations Between the *In vivo* Vascular and Metabolic Parameters

**Fig. 7:**
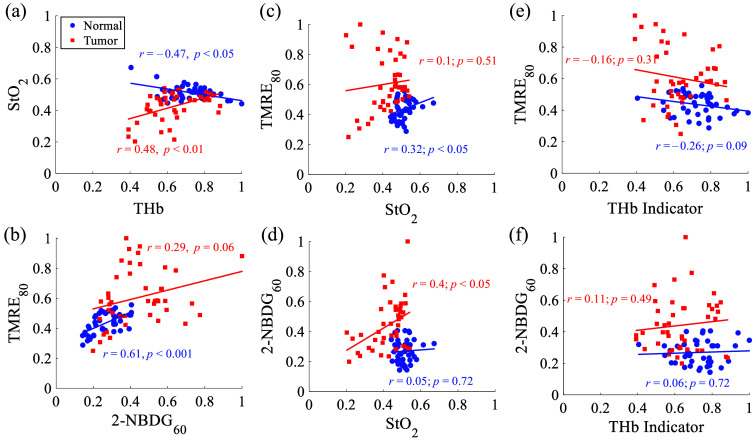
Correlations between vascular and metabolic parameters for normal tongue tissues and tongue tumor tissues include (a) ${\mathrm{StO}}_{2}$ versus THb indicator, (b) $2\text{-}{\mathrm{NBDG}}_{60}$ uptake versus ${\mathrm{TMRE}}_{80}$ uptake, (c) ${\mathrm{TMRE}}_{80}$ uptake versus ${\mathrm{StO}}_{2}$, (d) $2\text{-}{\mathrm{NBDG}}_{60}$ uptake versus ${\mathrm{StO}}_{2}$, (e) ${\mathrm{TMRE}}_{80}$ uptake versus THb indicator, and (f) $2\text{-}{\mathrm{NBDG}}_{60}$ uptake versus THb indicator. All metabolic values and THb indicators were normalized to their own global highest value, i.e., the highest $2\text{-}{\mathrm{NBDG}}_{60}$ and ${\mathrm{TMRE}}_{80}$ intensity across all normal and tumor tissues. Each animal image was divided into nine equal nonants, so we could present each animal’s data with a certain level of heterogeneity using nine mean values rather than just one single average value of these parameters. Images for all five normal tongue tissues and five tongue tumors were analyzed. Pearson’s correlation coefficients and p-values were calculated using the MATLAB (Mathworks, United States) statistics toolbox. The scale bar represents 400  μm.

The correlations between the vascular and metabolic parameters were analyzed for both normal tongue and tumor tongue tissues. A regional average approach, i.e., dividing each image into nine equal regions, was used as a practical way to explore the correlations among these parameter pairs. Specifically, each animal image was divided into nine equal nonants so each animal data will be reported with nine mean values rather than just one single average value of these parameters. Then, the correlation between these parameter pairs was explored based on the nine mean values of the equally divided regions per animal for all animals. Figure 7 shows the scatter plot and correlation analysis between six different parameter pairs including (A) ${\mathrm{StO}}_{2}$ versus THb indicator, (B) $2\text{-}{\mathrm{NBDG}}_{60}$ uptake versus ${\mathrm{TMRE}}_{80}$ uptake, (C) ${\mathrm{TMRE}}_{80}$ uptake versus ${\mathrm{StO}}_{2}$, (D) $2\text{-}{\mathrm{NBDG}}_{60}$ uptake versus ${\mathrm{StO}}_{2}$, (E) ${\mathrm{TMRE}}_{80}$ uptake versus THb indicator, and (F) $2\text{-}{\mathrm{NBDG}}_{60}$ uptake versus THb indicator. All metabolic values and THb indicators were normalized to their own global highest value, i.e., the highest THb, $2\text{-}{\mathrm{NBDG}}_{60}$, and ${\mathrm{TMRE}}_{80}$ intensity across all normal and tumor tissues. Figure 7(A) shows that normal tongue tissues and tongue tumor tissues had different correlations between ${\mathrm{StO}}_{2}$ and THb indicators. Specifically, tongue tumor tissues had a positive correlation (r=0.48, p<0.01) between ${\mathrm{StO}}_{2}$ and THb indicator, whereas normal tongue tissues had a negative correlation (r=−0.47, p<0.05) between the two parameters. Figure 7(B) shows that both normal tongue tissues and tongue tumor tissues had a positive correlation between ${\mathrm{TMRE}}_{80}$ and $2\text{-}{\mathrm{NBDG}}_{60}$, but only significant for normal tongue tissues (r=0.61, p<0.001). Figure 7(C) shows that normal tongue tissues had a positive correlation between ${\mathrm{TMRE}}_{80}$ and ${\mathrm{StO}}_{2}$ (r=0.32, p<0.05), whereas tongue tumor tissues had no obvious correlations between the two parameters (r=0.1, p=0.51). Figure 7(D) shows that normal tongue tissues had no obvious correlation between $2\text{-}{\mathrm{NBDG}}_{60}$ and ${\mathrm{StO}}_{2}$ (r=0.05, p<0.72), whereas tongue tumor tissues had a statistically significant positive correlation between the two parameters (r=0.4, p<0.05). Figures 7(E) and 7(F) show that there was no obvious correlation between the metabolic parameters ($2\text{-}{\mathrm{NBDG}}_{60}$ and ${\mathrm{TMRE}}_{80}$) and THb indicator for either normal tongue tissues or tongue tumor tissues.

## 4 Discussion

Many biological processes, such as tumor cell survival and recurrence, are selective and highly localized. The use of optical microscopy to capture spatial heterogeneity is vital for observing localized functional changes in tumors. These microscopic insights may reveal how cancer cells adapt to survive and potentially recur. Techniques such as fluorescence lifetime and Raman microscopy allow for in-depth visualization of biochemical processes, highlighting regions with distinct metabolic or structural profiles.^49^^,^^50^ This spatiotemporal data is essential in oncological research, as it informs targeted therapies by pinpointing resistant cell populations. Moreover, imaging multiple critical metabolic endpoints alongside vascular structures *in vivo* can deepen researchers’ understanding of the entire therapeutic landscape in cancer treatment.

In our previous studies, we demonstrated the use of an optical microscope with high-power lasers to simultaneously image MMP and glucose uptake *in vivo*.^39^ In addition, we have developed a label-free dark-field microscope to study tumor angiogenesis.^45^ To maximize the ease of and access to *in vivo* tissue metabolic imaging capability for cancer research, here we report a cost-effective, portable multi-parametric microscope to enable noninvasive metabolic and vascular imaging on biological models *in vivo* in near real time. To characterize the microscope system and image processing algorithms for vascular and metabolic parameters quantification, we have conducted various tissue-mimicking phantom studies. Our fluorescence phantom studies showed that the reported portable and cost-effective system had a comparable sensitivity compared with our former expensive benchtop microscope^39^ for the measurement of the two metabolic probes. Our hemoglobin phantoms studies showed that our portable microscope had a comparable accuracy compared with our well-established spectroscopy system for the estimations of ${\mathrm{StO}}_{2}$ and total hemoglobin contents.^43^ To demonstrate the capabilities of our device in capturing the dynamic and functional changes associated with HNC, we performed *in vivo* metabolic and vascular imaging of orthotopic tongue tumors in a preclinical model. Our preclinical results showed significantly increased glucose uptake and elevated MMP in tongue tumors compared with normal tongue tissues, along with reduced vascular oxygenation levels. Spatial analysis further highlighted a more heterogeneous distribution of metabolic activity and oxygenation within the tumor tissue.

The use of high-power dual-color LEDs (450SR-545SR, Prizmatix) instead of expensive lasers for both fluorescence and diffuse reflectance imaging has notably decreased costs (∼5 times) and reduced system size (∼3 times) compared with traditional benchtop microscopes. Dark-field-based diffuse reflectance imaging was collected by eliminating specular reflection using a pair of iris and a custom-cut mirror. The iris-mirror configuration maximized the rejection of specularly reflected light while ensuring sufficient signal capture. In addition, the use of quantitative analysis methods on spectral images allowed for effective vascular ${\mathrm{StO}}_{2}$ quantification. The modified Beer’s law illustrated in Eq. 1 has been implemented in microscope systems for ${\mathrm{StO}}_{2}$ estimation. Former window chamber imaging studies employing Eq. 1 have relied on the optical properties of normal skin tissue to estimate the $\mu_{\mathrm{eff}}$. This approach assumes that the effects of absorption and scattering in microscopic imaging are primarily confined to the superficial layer (a few hundred microns) above the tumor target, where the superficial layer has mostly normal skin tissue-like optical properties. Under this same concept, the $\mu_{\mathrm{eff}}$ used in Eq. 1 in our studies was estimated from normal mouse tongue tissue optical properties as reported previously.^43^ Our tissue-mimicking phantom studies showed that our microscope and the simple image processing algorithm can estimate ${\mathrm{StO}}_{2}$ values with a comparable accuracy compared with our benchtop spectroscopy platforms. However, it should be noted that the accuracy of the algorithm may be reduced in animal studies due to the assumption made in the use of the modified Beer’s Law for ${\mathrm{StO}}_{2}$ estimation. To improve the accuracy of the algorithm, one may need to know the absorption and scattering properties of the normal tissues and tumor tissues imaged by the microscope. This can be achieved using either a complicated hyperspectral microscope or a combined microscope (for imaging) and spectroscopy (for absorption and scattering extraction) platform, which will be explored in our future study plans.

The dark-field-based diffuse reflectance and fluorescence images were not acquired at the exact same time point in our current study but indeed acquired during the same experiment with about 1-h intervals to ensure a close temporal alignment. Some minor spatial variations between the two sets of images may occur due to the potential animal movements under anesthesia. In these cases, simple pixel shifting was used to adjust the reflectance images to match the corresponding fluorescence images, avoiding any minor misalignments that arose. Then, the simple pixel-shifting processed diffuse reflectance vessel mask was applied to the fluorescence image with minimal spatial variation to ensure that only the fluorescence in non-vessel regions was analyzed. We noticed that some small blood vessels were not visible in the dark-field-based diffuse reflectance images, likely due to the shallow penetration depth of visible light in microscopy. The tissue absorption primarily from the vessel regions can alter the fluorescence signal, leading to variations in the measured intensities. To minimize such influences on fluorescence signals, we have used spatial registration of the diffuse reflectance images to identify and exclude major blood vessel regions that may cause distortions in the fluorescence signal. To further minimize such influences from small vessel regions, we have further used fluorescence images to identify and exclude small blood vessels that may cause distortions in the fluorescence signals. However, we understand that this will not help remove the effect from regions that may have vessels but are not visible in either dark-field-based diffuse reflectance or fluorescence images, which is one limitation of our technique as it does not have the 3D imaging capability. To completely remove this effect, 3D imaging technologies may help to identify the vessels in deeper regions, then proper fluorescence corrections will be required to mitigate the associated distortions, which will be considered in our future work. We also noticed that the contrast in fluorescence images (shown in Fig. 5(A) was low in terms of vessel visualization, which may lead to low accuracy for vessel identification. Therefore, a tradeoff between excluding the small vessels and the potential errors in identifying the small errors from fluorescence images should be made for future studies. Given that the diffuse reflectance and fluorescence images were acquired 1 h apart, manual image co-registration and segmentation steps were involved in the image analysis. This manual procedure may (1) potentially introduce bias and (2) become practically challenging when large image data are to be processed. Therefore, more objective and efficient techniques such as machine learning-based segmentation and co-registration methods should be considered for future studies.

Hypoxia is crucial for tumor survival and recurrence, and optical imaging offers a label-free, non-destructive method to investigate its effects on tumors. Our extracted vascular parameters in Figure 4 indicate that tongue tumors exhibit significantly lower ${\mathrm{StO}}_{2}$ and ${\mathrm{HbO}}_{2}$ levels compared with normal tongue tissue, suggesting a greater prevalence of hypoxic regions within the tumors. These findings align with our previous spectroscopic studies on orthotopic tongue tumors.^43^ Our former study revealed a greater difference between the two groups, likely due to spectroscopy’s ability to provide volumetric information. However, optical imaging can generate a visual vessel map, enabling the observation of the heterogeneity in vascular oxygenation distribution. This capability provides insights into how different regions within the tumor may experience varying levels of oxygenation, further elucidating the relationship between tumor vascularization and hypoxia.

Our metabolic studies revealed distinct metabolic demands in tongue tumors compared with normal tongue tissues in Fig. 5. We observed significantly higher levels of both $2\text{-}{\mathrm{NBDG}}_{60}$ (indicating glucose uptake) and ${\mathrm{TMRE}}_{80}$ (measuring MMP) in the tongue tumors compared with normal tongue tissues. These increased glycolytic and mitochondrial activities suggest that tongue tumors have an increased metabolic activity, which may contribute to their growth and survival. Optical imaging has enabled further to visualize metabolic trends spatially, allowing for a more detailed analysis of phenotypes. The local range variation analysis in Fig. 6 reveals higher local range values in tongue tumors compared with normal tongue tissue. This indicates increased metabolic heterogeneity in regions with higher metabolic demand, suggesting that certain areas within the tumors may be more metabolically active. Figure 6(C2) further illustrates that tongue tumors have a higher number of pixels where either glycolytic ($2\text{-}{\mathrm{NBDG}}_{60}$) or mitochondrial (${\mathrm{TMRE}}_{80}$) activity is high compared with normal tongue tissue. This finding underscores the enhanced metabolic heterogeneity in tumor regions, indicating a greater reliance on both glycolysis and mitochondrial function in sustaining tumor growth.

In previous preclinical metabolic measurements on small animals, an optimized 6-h fasting procedure has been routinely used to minimize the variance in metabolic demand among the animals.^33^ This fasting procedure minimizes variability in metabolism caused by recent food intake and provides a controlled baseline for assessing metabolic function, thereby one can ensure that the measured metabolic differences were primarily attributable to the biological condition under study (e.g., normal vs. tumor) rather than variations in animal feeding behavior. However, the potential implications of this fasting procedure include physiological stress in small animals and the possibility of not capturing inherent metabolic changes under non-fasting conditions. In this pilot study, we primarily focused on demonstrating the capability of our portable microscope to image the vascular (${\mathrm{StO}}_{2}$, THb indicator) and metabolic parameters (2-NBDG and TMRE) on the same tissue site with about one hour delay *in vivo* using an orthotopic tongue model. This study involved monitoring the animals for 80 min to capture the kinetics curves for 2-NBDG and TMRE uptake as shown in Figs. 5(B)–5(C). It should be noted that the noninvasive nature of our technique allows for repeated measurements and longitudinal studies as needed. In our future studies, we plan to leverage this technology for longitudinal monitoring of these key endpoints under various treatment responses for cancer therapeutics discovery. Both 2-NBDG and TMRE have been thoroughly validated in previous studies for the measurement of glucose uptake and mitochondrial function. Specifically, 2-NBDG has been validated against FDG *in vitro* and *in vivo*,^51^ whereas TMRE has been extensively validated both *in vitro* and *in vivo*.^32^^,^^36^^–^^38^ Therefore, the validation of the two metabolic probes was not repeated in this study given our focus was to demonstrate a portable, cost-effective multi-parametric optical microscopy for functional imaging of small tongue tumors *in vivo*.

Preclinical xenograft tumor models including the commonly used flank tumor models^52^ and optically thin window chamber models^47^ have been developed and utilized to study the complex tumor microenvironment and tumor therapeutics responses. In these subcutaneous tumor models, cancer cells or tumor tissues are implanted subcutaneously to form a small tumor that allows one to study cancer biology or therapeutic responses *in vivo*. Orthotopic tumor models, implanting cancer cells or tumor chunks in a region where a particular cancer normally arises, have been developed to provide a more realistic model for cancer inquiries compared with the subcutaneous tumor models.^43^ In this study, we established and utilized the orthotopic tongue cancer model for oral cancer research. The orthotopic oral cancer models allow for more accurate predictions of metastatic potential, a better representation of tumor heterogeneity, and significantly enhance the translational relevance of findings to human diseases. The orthotopic tongue tumor model of HNC used in this study provides significant translational relevance by closely mimicking the native tumor microenvironment (TME). However, it should be noted that its applicability to clinical settings could be limited by the inherent differences between animal models and human patients, including variations in immune system dynamics, tumor growth patterns, and more complicated physiological structures. Though we demonstrated our portable optical platform for oral cancer study using tongue cancer models in this study, our platform can be used on other tumor models such as optically thin window chamber models where the optical penetration will not be a big concern, for various types of therapeutic responses study (chemo- or targeted-therapies). However, it should be noted that the portable multi-parametric microscopy imaging techniques demonstrated in this study also pose certain technical limitations that should be considered in future studies. For instance, (1) the fluorescence images may be prone to distortions caused by tissue absorption and scattering, which require careful fluorescence corrections; (2) the microscope has no sectioning capability, which restricts its ability to capture signals from deeper regions; and (3) the imaging processing algorithm was off-line which may not meet the real-time imaging requirement in a clinical setting. It should be noted that tissue absorption and scattering caused distortions on fluorescence images is one of the critical and significant challenges in the fluorescence-based functional microscopy field, which is also one limitation of our current study that will be addressed in our future study. Our former studies^43^ reported that normal mouse tongue tissues and small tongue tumor tissues had comparably reduced scattering coefficients. Moreover, we primarily analyzed the fluorescence in the non-vessel region (with minimal absorption) to report metabolic parameters in our current study. Therefore, we believe that the metabolic differences between normal tongue tissues and tongue tumor tissue reported in this study will not change significantly with or without consideration of the tissue absorption and scattering distortions. However, careful fluorescence correction will be necessary to report metabolic parameters of tissue types that have significantly different absorption and scattering coefficients, which will be explored in our future studies.

Our new platform has a comparable resolution (∼3.5  μm) and FOV ($∼2.5\;{\mathrm{mm}}^{2}$) compared with our previously published benchtop microscope^39^ when the same objective lens was used, but the new platform has significantly improved portability (∼3 times) and reduced costs (∼5 times) as illustrated in Fig. 1. The traditional microscopes such as two-photon or confocal have a higher resolution (subcellular) but with very limited FOV (few hundred microns),^53^ the hyperspectral imaging platforms have a wide FOV (centimeters) but with a lower resolution (∼20  μm).^36^ Our portable microscope will fill the technical gap between the traditional microscopy platforms, allowing one to capture a tumor’s metabolism within its vascular microenvironment *in vivo* with a microscope-level resolution and millimeter FOV for tumor biology study. With the use of a 4× objective lens, the resolution of our microscope was measured to be ∼3.5  μm using a resolution target. However, it should be noted that the actual resolution of the system in tissue imaging could be reduced due to tissue scattering. Nevertheless, our vascular and metabolic images for all normal tongue tissues and tongue tumor tissues shown in the study demonstrated that our microscope was able to capture relatively smaller vessels (∼10  μm) in addition to the big blood vessels. One can easily switch the 4× objective lens with a 10× or 20× objective for a better resolution but with a sacrifice of field of view. Our fluorescence phantom studies showed that our microscope with a compact CCD camera (CS505MU, Thorlabs) can measure the two metabolic probes at biologically low concentrations with a wide dynamic range (0 to 8  μM for 2-NBDG and 0 to 70 nM for TMRE), which is comparable with our formerly reported laser-based benchtop microscope. However, if a higher sensitivity or wider dynamic range is required in future studies, a more sensitive scientific camera should be considered.

Capturing vascular and metabolic endpoints on the same tissue site enabled us to explore the spatial correlation of these parameters more comprehensively. These spatial correlations reveal that tumor tissue deviates significantly from the normal group. For instance, Fig. 7 showed that normal tissue had a positive correlation between TMRE and ${\mathrm{StO}}_{2}$ (p<0.05) and a positive correlation between TMRE and 2-NBDG (p<0.001). These correlations suggested that normal tongue tissues primarily rely on mitochondrial metabolism that is dependent on both oxygen levels and glucose uptake, which is well supported by former studies.^54^ By contrast, tongue tumor tissues had a positive correlation between 2-NBDG and ${\mathrm{StO}}_{2}$ (p<0.05) and a marginally positive correlation (p=0.06) between TMRE and 2-NBDG. These correlations suggest that the small tongue tumors studied here primarily rely on aerobic respiration for energy production.^54^ Small tongue tumors still have active mitochondria but are not necessarily oxygen-dependent.^54^ These different correlations clearly showed that tongue tumors may have different metabolic pathways compared with normal tissues. Generally, it is expected that a higher THb (blood supply) may lead to a higher ${\mathrm{StO}}_{2}$, which was observed in the small tongue tumors with a positive correlation between ${\mathrm{StO}}_{2}$ and THb indicators (p<0.01). However, our study captured that the normal tongue tissues had a negative correlation between the two vascular parameters (p<0.05). Given there were minimal differences in ${\mathrm{StO}}_{2}$ values for normal tongue tissues in this analysis, this unexpected negative correlation may not be physiologically significant. Our correlation study shows the potential of optical imaging can enhance our understanding of the interplay between oxygenation and metabolic activity within the tumor microenvironment. These preliminary findings underscore the potential of our multi-parametric microscope as an effective, accessible tool for both unveiling critical aspects of tumor progression and informing therapeutic response strategies. In future work, we aim to implement our empirical radiometric techniques with our microscope to quantify metabolic parameters more accurately by correcting the distortion in fluorescence caused by absorption and scattering.^55^

The potential influence of surface irregularities is a limitation of this current study, particularly in tumor-bearing mouse tongues, which are less flat compared with normal tongues. Although we minimized this issue by attaching the tongues to a flat platform and limiting tumor size to ∼3  mm, some surface irregularities remained. To mitigate this, we also normalized the dark-field-based diffuse reflectance and fluorescence signals of both normal and tumor tongues using a reflective standard and a flat fluorescence slide as references. Despite these efforts, complete compensation for surface irregularities may be challenging, which has been encountered in other imaging studies using mammary pad models and window chamber models. Future improvements could involve the integration of tissue curvature correction algorithms, which are beyond the current scope of our study but represent a promising area for enhancing imaging accuracy in similar experimental models.

## 5 Conclusion

We have reported a portable, multi-parametric functional microscope that enables noninvasive imaging of critical vascular and metabolic parameters in biological tissues in near real-time. Using an orthotopic tongue tumor model, we successfully conducted near-simultaneous imaging of vascular and metabolic parameters *in vivo* for cancer biology study. Our microscopy platform holds the potential to offer new opportunities for cancer discoveries with detailed insights into tumor metabolism reprogramming and potential therapeutic responses.

## Data Availability

Data underlying the results presented in this paper are not publicly available at this time but may be obtained from the authors upon reasonable request.
